# Recent Progress in the Detection and Monitoring of Toxin-Producing Cyanoprokaryotes and Their Toxins

**DOI:** 10.3390/toxics14010086

**Published:** 2026-01-18

**Authors:** Milena Pasheva, Milka Nashar, Diana Ivanova

**Affiliations:** Department of Biochemistry, Molecular Medicine and Nutrigenomics, Faculty of Pharmacy, Medical University of Varna, 9002 Varna, Bulgaria; milka.nashar@mu-varna.bg (M.N.); divanova@mu-varna.bg (D.I.)

**Keywords:** cyanobacteria, cyanotoxins, early detection methods, harmful algal blooms

## Abstract

Eutrophication of water bodies and the bloom of toxin-producing cyanoprokaryotes raise health concerns. Various cyanoprokaryotes species, including *Microcystis*, *Raphidiopsis*, *Nodularia*, and *Chrysosporum*, release toxins into the aquatic environment, which can reach concentrations toxic to humans and animals. Rising temperatures and human activities are primary drivers behind the increasing frequency of toxic cyanobacterial blooms. The Word Health Organization (WHO) has established provisional guideline values for cyanotoxins in drinking water and water used for other purposes in daily human activities, and has published guidance for identifying hazards and managing risks posed by cyanobacteria and their toxins. There are currently no acceptable limit values for cyanotoxins. To address monitoring needs, contemporary strategies now incorporate molecular genetics, immunoassays, biochemical profiling, and emerging machine-learning frameworks. This paper reviews current early detection methods for harmful cyanobacterial blooms, highlighting their practical advantages and drawbacks.

## 1. Introduction

Cyanoprokaryotes are unicellular organisms, among the most ancient inhabitants of the Earth, commonly known as blue-green algae. They are known for their ability to cause water “blooms” and release toxins (cyanotoxins). Their massive development, together with the ability of certain species or their strains to produce toxins under conditions of current climate change and increased anthropogenic pressure, turns them into a new risk factor for human and ecosystem health [1]. The growing interest in the study of harmful bloom-forming cyanoprokaryotes stimulates toxicological, pharmacological, and ecological scientific research focused on potentially toxic species and their hazardous products [2]. Managing the health risks of cyanobacteria has become more systematic through the World Health Organization’s provisional guideline values and hazard identification manuals for cyanotoxins in drinking water and water used for other purposes in daily human activities [3]. According to Annex II of the Water Framework Directive 2000/60/EC, transposed into Bulgarian legislation for the characterization of surface waters, there are currently no acceptable limit values for cyanotoxins. A study of the cyanoprokaryote blooms in Bulgaria (Projects No KP-06-OPR03/18, 19.02.2018 funded by the Science Research Fund of the Ministry of Education and Science) encountered a problem in identifying the most appropriate methods for the detection and monitoring of toxin-producing cyanoprokaryotes and their toxins. The detection and quantification of cyanoprokaryotes and their associated toxins present a significant methodological challenge, as the choice of the best analysis is related to its specificity with regard to different taxonomic species, sensitivity, and the range of quantitative detection of the main groups of cyanotoxins: microcystins (MC), cylindrospermopsins (CYN), nodularins (NOD), saxitoxin (STX), and anatoxin-a (ATX). Furthermore, our understanding of cyanobacteria is geographically skewed, with the majority of data originating from freshwater bodies in China and the West. This leaves a significant gap in our knowledge of understudied habitats, where toxic metabolites present both a public health threat and a resource for biotechnology. Shifting research focus to these neglected areas is vital for uncovering the true extent of cyanobacterial variety and its environmental impacts. [4,5].

This review places primary emphasis on the most widely applied methods for the detection and monitoring of toxin-producing cyanoprokaryotes and their toxins in biological samples and waters.

## 2. Detection and Quantification of Cyanoprokaryotes and Their Toxins

Two major approaches are applied: direct analysis of either the presence of cyanoprokaryotes or of their toxins in natural and biological samples and indirect detection of possible presence, inferred from established, likely induced, and expected effects of cyanoprokaryotes or their toxins on isolated cells or living organisms. Each method demonstrates specific limitations regarding sensitivity, reliability, and detection limits [6]. In vivo and in vitro studies are gaining increasing popularity, as they track the metabolic and biological effects of algae toxins or their producers in the context of the ecosystem changes they provoke, while the taxonomical identification of cyanotoxin-producing prokaryotes requires special qualifications of specialists in lower plants, and remains quite challenging.

### 2.1. Direct Microscopy

Different microscopic techniques for cyanobacteria bloom sample examination have been applied and the classical light microscopy at a magnification of 100–400 times is the first choice and a standard method. Along with that, other microscopy techniques are used for more specific goals, such as determination of morphology specificity or ecological features of cyanobacteria—scanning electron microscopy (SEM), confocal laser scanning microscopy CLSM), and transmission electron microscopy (TEM) [7]. Direct microscopy is really useful, as the information on the species present in the water bloom sample may be used to predict the type of cyanotoxins that may be present and thus suggest the subsequent method for toxin analysis. On the other hand, morphological characteristics do not completely correspond to genotypes of environmental populations. Thus, organisms identified as belonging to the same species may express different types and quantities of toxins, which makes predictions about toxins uncertain. [8]. An integrative analysis of cyanobacterial communities in waterbodies of Moscow, Russia, established high convergence between microscopy and rRNA-based metabarcoding data for dominant genera, ascertaining the efficiency of metabarcoding as a rapid and reliable tool for detection of rare taxa missed by microscopy of harmful water cyanobacterial bloom samples. The method, however, is limited to distinguishing between toxigenic and nontoxigenic species [9].

Standard and inverted microscopes are also used for cyanobacteria quantification. An inverted microscope with counting chambers is a general preferred approach for estimating cyanobacterial numbers, while the standard microscope is sufficient for preconcentrated or naturally dense samples when the volume of the sample can be precisely defined [8].

At the same time, the traditional single-technique monitoring can easily miss episodic and transient cyanobacteria harmful algal-bloom events, as cyanobacterial biomass may vary between samples taken within a few hours or within short distances [10]. Analysis of a single sample, even though accurate, may not be indicative to assess the population size, thus imposing the requirement for multiple sampling, validation of methods, and integration of new molecular methods to control the results of the classical direct microscopy.

### 2.2. Molecular-Genetic Methods

The high specificity, reliability, and rapid turnaround of molecular assays provide a robust framework for quantifying cyanobacteria and identifying the presence of toxin-encoding genes in environmental samples. Due to their low detection limits, molecular-genetic assays facilitate the early identification of toxigenic strains, providing a crucial lead time for public health responses before blooms become visible.

#### 2.2.1. Conventional Polymerase Chain Reaction (PCR)

The method consists of in vitro multiplication (amplification) of a selected gene from template DNA using a pair of primers. After repeated cycles of heating and cooling, through the activity of a thermostable DNA polymerase, a high amount of DNA containing the nucleotide sequence of interest is obtained. The genes responsible for the production of cyanotoxins can be detected through sequencing of the cyanobacterial genome, which makes possible the PCR amplification of a target gene [11]. In the context of cyanobacteria, these molecular techniques serve as a definitive diagnostic tool for confirming their presence within environmental water samples. This is usually achieved by amplifying the gene for 16S ribosomal RNA, which can easily be visualized on an agarose gel [12].

#### 2.2.2. Multiplex Polymerase CHAIN reaction (mPCR)

A method has been introduced that simultaneously identifies different taxonomic genetic markers associated with cylindrospermopsin production. Specifically, targeting the constitutive *rpoC1* gene allows for the differentiation of *Cylindrospermopsis raciborskii* from other toxin-producing species like *Anabaena bergii* and *Aphanizomenon ovalisporum* [13]. Furthermore, Valério et al. [8] enhanced diagnostic capabilities by developing a multiplex PCR method that simultaneously amplifies the *mcyA-cd*, *mcyAB*, and *mcyB* fragments of the microcystin gene cluster. To validate the multiplex PCR, high-performance liquid chromatography (HPLC) was employed as the “gold standard” for toxicological assessment; the PCR assay demonstrated a sensitivity of 92.3% and a specificity of 100%. In this context, multiplex PCR can be considered a valid method for immediate indication of the cyanobacterial bloom’s toxic potential, providing useful information for monitoring water bodies by local authorities [6]. In a large-scale application of these markers, Moreira et al. [14] analyzed freshwater basins for the four primary cyanotoxins. Their findings revealed a high prevalence of toxin-encoding genes: 79% for microcystins (*mcyA*), 50% for saxitoxins (*sxtI*), and 40% each for cylindrospermopsins (*cyrC*) and anatoxins (*anaC*).

#### 2.2.3. Real-Time Polymerase Chain Reaction (Real-Time PCR)

In this method, the amplification process can be monitored in real time through fluorescent labeling using TaqMan probes. A study by Rinta-Kanto et al. [15] aimed at detecting cyanotoxins in samples from Lake Erie in Oregon was conducted using real-time PCR analysis with specific TaqMan probes targeting a conserved microcystin-specific 16S rRNA fragment and the microcystin synthetase gene mcyD. Real-time PCR primers targeting the $rpoC1$ gene fragment were developed by Churro et al. [16] for *Planktothrix agardhii*; the resulting cell concentrations demonstrated a strong linear relationship with traditional microscopic counting methods. These findings establish the method as a precise, cost-efficient tool for the rapid, absolute quantification of potentially toxic cyanobacteria. However, due to various technical and biological factors, the determination of the actual toxin concentration can be inconsistent [17]. The quantitative polymerase chain reaction (qPCR) method has been applied for detecting cyanobacteria (via the 16S rRNA gene) and genes responsible for the synthesis of microcystins, cylindrospermopsins, nodularins, and saxitoxins in water samples from Lake Karaoun, collected during 2019–2020. Fifty percent of the samples tested positive for MC-producing genes (mcyE). Analysis revealed the presence of eleven microcystin (MC) variants, with peak concentrations reaching 211 μg/L for MC-LR and 199 μg/L for MC-YR. The ‘LR’ and ‘YR’ designations refer to the variable amino acids within the toxin’s cyclic structure—specifically leucine (L), arginine (R) and tyrosine (Y). In contrast, molecular screening for cylindrospermopsin (*cyrA*) and saxitoxin (*stxA*) production genes yielded no detections across any of the analyzed samples [18]. Groundbreaking research by Žegura et al. [19] utilized quantitative real-time PCR to provide the first evidence that cylindrospermopsin (CYN) at a concentration of 0.5 μg/mL modulates gene expression within human peripheral blood lymphocytes (HPBLs). Exposure induced the expression of genes presumed to participate in CYN metabolism (CYP1A1 and CYP1A2). Exposure to CYN was found to alter the mRNA expression of the p53 protein alongside its downstream targets—specifically *MDM2* and *GADD45α*—which are critical mediators of the DNA damage response and apoptotic pathways. These findings provide convincing evidence that CYN should be considered genotoxic and that lymphocytes may also be a target of cylindrospermopsin-induced genotoxicity [14]. Conversely, in human liver cancer cell lines (HepG2), CYN did not induce any change in p53 tumor-suppressor gene expression at any of the tested concentrations after 4 and 24 h of exposure Štraser et al. [20], whereas Bain et al. [21] detected accumulation of p53 protein in the same cell line only after a longer exposure of 48 h. mRNA levels of selected genes were measured after treatment of human embryonic kidney 293 cells (HEK293) with 0.5 and 5 μg/mL CYN for 4 and 24 h. The results showed upregulation of gene expression after the longer exposure (24 h) and the highest concentration (5 μg/mL). Targeted genes spanned several functional categories, including xenobiotic metabolism (CYP1A1 and CYP1A2), DNA damage response (TP53 and CDKN1A), oxidative stress (CAT and GPX1), and apoptosis (BCL2). Exposure to a lower concentration (0.5 μg/mL) altered the expression of CYP1A2, CDKN1A, and GPX1 within 24 h. Notably, the apoptosis-related gene *BAX* was the only marker that exhibited no change in expression [22]. Lu et al. [23] tested both qPCR and reverse transcription qPCR (RT-qPCR). RT-qPCR was included to assess toxin gene expression in comparison to toxin levels. The authors found that detection of mcyA via qPCR or RT-qPCR increased approximately three weeks before increases in microcystins were observed through enzyme-linked immunosorbent assay (ELISA). Consequently, qPCR is an effective method for identifying the early indicators of potential harmful cyanobacterial outbreaks.

### 2.3. Biochemical Methods

These types of analysis represent easy and accessible approaches for the rapid determination of cyanotoxin concentration. These detection methods work alongside molecular-biological approaches to verify the presence and concentration of toxins in aquatic environments.

#### 2.3.1. Enzyme-Linked Immunosorbent Assay (ELISA)

This approach exploits specific antibody–antigen interactions, to enable the sensitive detection of low-level proteins, peptides, and hormones in liquid samples. ELISA offers a highly specific and sensitive detection platform with the benefit of rapid analysis. This assay is useful for screening, with detection limits around 0.1 μg/L. Metcalf et al. [24] obtained polyclonal antibodies and developed an indirect competitive immunoassay showing good reactivity against one of the microcystin variants (MC-LR), which can be successfully used for determining MC content in environmental samples. Results from the ELISA analysis of water samples conducted by Sheng et al. [25] demonstrated a quantitative detection range for MC-LR from 0.17 to 2.32 μg/L, allowing the authors to conclude that the method is sensitive and reliable for detecting cyanotoxin contamination in water bodies and drinking water. ELISA has also been successfully applied to the detection of cyanotoxins in benthic mats. For instance, Bauer et al. [26] employed this method to quantify saxitoxin levels in the Lech River (Germany) following a series of canine poisoning incidents. Their analysis revealed peak saxitoxin concentrations of 0.59 μg/L within benthic mats and 0.35 μg/L in the surrounding open water. Using this assay, microcystins have been detected in dead mallard ducks (Foss et al. [27]), mussels (*Mytilus galloprovincialis*) (Baralla et al. [28]), and freshwater fish and marine mussels (Preece et al. [29]). Moreover, this technique is capable of detecting covalently bound MC, which is an advantage because microcystins can bind covalently with the catalytic subunits of protein phosphatases 1 and 2A, which are primarily found in liver tissue [30]. Table 1 provides a summary of the ELISA methods used for detecting microcystin, nodularin, and saxitoxin.

#### 2.3.2. Fluorescence Polarization Immunoassay (FPIA)

The principle of FPIA relies on the divergence in fluorescent polarization states between the labeled antigen and the larger, more slowly rotating labeled analyte–antibody complex. The FPIA assay operates on a competitive binding principle: when the target cyanotoxin concentration is significantly lower than that of the fluorescently labeled marker, the antibody predominantly binds the marker, yielding high fluorescence polarization (FP) values. In contrast, higher toxin concentrations in the sample displace the marker, resulting in decreased FP values. Under optimal conditions, this method provides detection limits of 0.86mg/L for MC-LR and 0.95 mg/L for NOD. These sensitivities are sufficient to meet the WHO’s recommended interim guidelines for monitoring water safety [38]. Lei et al. [39] used labeled secondary antibodies to detect MC-LR in water sources with a wide quantitative range between 0.01 and 10 ng/mL, confirming that the method is among the most sensitive for detecting microcystins. Xu et al. [40] developed a fluorescent enzyme-linked immunoassay without fluorescent nanoparticle labeling for ultrasensitive detection of microcystin-LR in water and fish samples. Under optimal conditions, this method provides a low detection limit of 0.6 ng/L, approximately 30 times lower than traditional indirect competitive ELISA. A rapid immunochromatographic assay was applied to detect microcystins in water samples from different sites of the Curonian Lagoon, collected between May and November 2017. For the first time, during the vegetation season, toxic metabolites were present across the entire southern lagoon coastline, though none were found in May. Peak concentrations of 5–10 µg/L occurred in late June and September, while all other samples yielded results below 2.5 µg/L or below the limit of detection [41]. For the first time, a multiplex immunochromatographic assay based on two-color fluorescent microspheres was developed. Under optimal conditions, the assay is completed in 20 min and reaches simultaneous detection limits for MC-LR (0.074 μg/kg) and okadaic acid (2.42 μg/kg) in fish samples [42]. Later, Cevallos-Cedeño et al. [43] optimized the immunochromatographic assay with both visual and instrumental result reading. This test showed a visual detection limit for anatoxin-a in water samples of 4 ng/mL. A multiplex immunochromatographic test system was developed for concurrent detection of three distinct aquatic toxins: domoic acid (DA), okadaic acid (OA), and microcystin-LR. A competitive indirect immunochromatographic assay was performed using gold-labeled secondary antibodies. The detection limits/working ranges were 0.05–0.29, 1.3–58.2, and 0.1–1.1 ng/mL for MC-LR, DA, and OA, respectively. With an assay time of just 18 min, this method represents a promising tool for the rapid, simple, and sensitive monitoring of both aqueous- and food-matrix safety [44].

#### 2.3.3. Protein Phosphatase Inhibitory Assay (PPIA)

Inhibition of eukaryotic protein phosphatases (PP) is a simple, fast, and sensitive colorimetric method. By measuring the absorbance at a target wavelength, the substrate or its resulting product can be quantified to assess enzyme activity, which correlates inversely with the toxin concentration. Depending on the protocol used, PPIA can detect toxins within several hours and is suitable for processing large numbers of samples. This procedure enables quantitative determination of MC-LR with a detection limit as low as 0.01 μg/L [45]. Measurements can be performed using radioisotope techniques based on 32P-labeled substrates, as well as colorimetric assays using substrates such as p-nitrophenyl phosphate. The assay features a working range of approximately 0.2–1 μg/L, enabling the direct quantification of cyanobacterial hepatotoxins in water samples without the need for prior concentration [46]. In a comparative study, Covaci et al. [47] evaluated the inhibitory effects of native PP2A, recombinant PP2A, and recombinant PP1 against three microcystin variants, MC-LR, MC-YR, and MC-RR. Their results identified MC-LR as the most potent inhibitor, followed by MC-YR and MC-RR. Among the enzymes tested, recombinant PP2A demonstrated the highest sensitivity to inhibition, surpassing both recombinant PP1 and native PP2A. Despite this sensitivity, a key limitation of the PPIA is its inability to distinguish between microcystins and nodularins. Therefore, results are often expressed as MC-LR equivalents. Additionally, when analyzing water containing cyanobacterial blooms, interference from unknown compounds must be considered, as it can lead to overestimation or underestimation of toxin concentrations [48]. Another drawback is the lack of specificity toward different MC variants, meaning that toxin identification is not possible and supplementary analyses are required to detect other cyanotoxins potentially present in the sample [49]. Without purification and isolation of MCs, the test also cannot differentiate cyanobacterial toxins from other environmental protein phosphatase inhibitors, such as okadaic acid, calyculin A, and tautomycin [50].

#### 2.3.4. High-Performance Liquid Chromatography (HPLC)

Liquid chromatography is an ideal method for the quantification of algotoxins, as it offers high sensitivity and specificity. It allows reliable measurement of multiple toxins in complex environmental and biological samples at very low ng/L concentrations. The use of the method implies a lower sample volume required for analysis, thus facilitating collection and storage activities [51].

Additional HPLC analysis in the study by Kezlya et al. [9] demonstrates that the presence of microcystins accounts for a high proportion (20–28%) of extremely toxic microcystin-leucine arginine compounds. A recent study in Bulgaria used aerial drone observations for the first time as an additional tool for selecting sampling points during blooms in selected water bodies. The use of HPLC analysis of marker pigments for rapid determination of phytoplankton composition and biomass allowed the detection of cylindrospermopsin in freshwater water bodies, the detection of saxitoxins in Lake Durankulak, and the first data on microcystins for the Blue River Dam [52]. Liquid Chromatography–Mass Spectrometry analysis (LC-MS) is a chemical technique in analytical chemistry that combines the physical separation capabilities of liquid chromatography (HPLC) with the mass analysis capabilities of mass spectrometry, allowing the detection and identification of chemicals in complex mixtures with high sensitivity and selectivity. This method offers the identification or confirmation of each individual component in the analyte. This method is optimized for detecting the ratio of cyanotoxins to microcystins, providing high specificity in the discrimination of structurally similar molecules [53].

The analysis of cyanotoxins by LC–MS requires precise instrumental operation, given the high sensitivity of the method. In addition, accurate identification and quantitative determination require certified reference standards for each compound to be analyzed, and it should be emphasized that standards are lacking for many types of cyanotoxins [54]. Different chromatographic methods exhibit higher sensitivity and accuracy than other immunological assays. A drawback is their high cost, which makes them unsuitable for widespread use in early bloom detection processes. Nevertheless, liquid chromatography–mass spectrometry is used for the quantitative determination of cyanoprokaryotes and serves as a reference for comparing the accuracy and precision of other methods in use [55]. On the other hand, the ultra-high-performance liquid chromatography (UHPLC) method can be used to demonstrate the content of active compounds, for example carotenoids, in various biological species, including the green alga *Caulerpa racemosa*, as was done in the study of [56]. Interestingly, the isolated carotenoid extract from *Caulerpa racemosa* exhibits good biological activity by inhibiting pro-inflammatory cytokines such as TNF-α, antioxidant activity proven by DPPH and the ABTS method, and inhibition of α-glucosidase, α-amylase, which makes it a potential for the development of pharmaceutical products.

### 2.4. Measurement of Biochemical Markers (Indirect Assays)

Toxic cyanobacteria can induce the production of reactive oxygen species (ROS), which leads to oxidative stress and consequently alters the antioxidant defense systems of organisms [57]. Enzymes such as superoxide dismutase (SOD) and catalase (CAT), as well as other molecules like lipoic and dihydrolipoic acid, are key components of antioxidant defense. Their increased activity in animal tissues plays an important role in eliminating excessive levels of ROS [58]. Suppression of glutathione S-transferase (GST) activity may result from inhibition of enzyme synthesis, due to high accumulation of MC during exposure and depletion of reduced glutathione as a response to MC toxicity [57]. A 20-day exposure to saxitoxins has shown that in fish liver SOD activity decreases in the treated group compared to the control, while GPX activity and glutathione (GSH) levels increase in the saxitoxin-exposed group. These findings indicate that antioxidant defenses are stimulated in response to toxin-induced oxidative stress [59]. Pham et al. [60] examined microcystin accumulation and changes in the activity of antioxidant enzymes CAT, SOD, and GST in edible mussels Corbicula leana, following 10-day exposure to 100 μg MC. The mussels accumulated up to 12.7 μg/g dry weight of free MC and 4.2 μg/g dry weight of covalently bound MC. The results indicate that consumption of mussels harvested during cyanobacterial blooms poses a significant health risk. Biological indicators were also studied in the serum of people living near a lake with recurrent cyanobacterial blooms who were chronically exposed to cyanotoxins, particularly microcystins. The study revealed increased levels of liver enzymes aspartate aminotransferase (AST) and alanine aminotransferase (ALT) in 4% and 6% of subjects, respectively, while elevated AST and ALT levels were observed in 11% and 13% of the population. A significant difference was found between exposed children and adults. The fact that adults showed greater deviations from reference values supports the idea that the longer the exposure to the toxin, the more severely the liver is affected, and therefore greater deviations in liver enzyme levels can be expected [61].

### 2.5. In Vivo Analyses

The mouse bioassay developed by Falconer [62] serves as a semi-quantitative approach, facilitating a comparison of lesion severity against control groups exposed to standardized concentrations of toxins, typically MC-LR. The experiment involves injecting a sample into a group of animals followed by autopsy after 24 h of exposure. An increase in liver weight and volume is recorded as an indicator of hepatocellular damage and the presence of hepatotoxins in the organism. One of the earliest studies, by Vasconcelos [63], showed that several species of wild fish from Portuguese freshwater sources could accumulate MC. However, the toxin levels detected in edible tissues did not suggest a risk to human health. Research on cyanobacterial effects in fish primarily focuses on the neurotoxin anatoxin-a (ATX). Results indicate that ATX produced by *Anabaena* sp. can induce adverse physiological effects and even mortality during early developmental stages of common carp (*Cyprinus carpio*) [64,65]. An in vivo study reported characteristic signs of renal toxicity, including necrosis, enlarged proximal tubule lumens, and glomerular alterations in mice treated with cylindrospermopsin [66]. Over the last decade, research interest has increased regarding the effects of cyanobacterial toxins on cultivated plants due to the use of contaminated water in agriculture. The effects of MC and CYN exposure on edible plants have been documented for rice (*Oryza sativa*) (Azevedo et al. [67]), carrot (*Daucus carota*) (Machado et al. [68]), lettuce (*Lactuca sativa*) (Pereira et al. [69]), and spinach (*Spinacea oleracea*) (Freitas et al. [70]). Documented effects on vegetation include inhibited plant growth, disrupted oxidative homeostasis, and diminished nutritional quality. A recent study by Llana-Ruiz-Cabello et al. [71] demonstrated that spinach exhibits greater sensitivity to both MC and CYN—whether administered individually or as a mixture—than lettuce. Furthermore, plants exposed to the toxin combination showed distinct accumulation patterns, with CYN being assimilated more readily than MC.

### 2.6. In Vitro Analyses

Treatment of cells with a cytotoxic compound can lead to different cellular responses. Cells may undergo necrosis, losing membrane integrity and dying as a result of cell lysis. They may cease to grow and divide, or they may activate a genetic program for controlled cell death (apoptosis). A study by Dias et al. [72] evaluated the cytotoxicity of MC-LR on a renal cell line (Vero-E6). After 72 h of exposure to extracts from Microcystis aeruginosa producing MC-LR, cytotoxic effects were recorded in a concentrations ranging from 11 to 100 μM, with results varying based on the specific viability assay employed. These findings indicate that the observed cytotoxicity of the *M. aeruginosa* extract are attributable to MC-LR, rather than other secondary bioactive cyanobacterial metabolites. Given that MC-LR is recognized as a potent tumor promoter, Dias et al. [73] further investigated whether exposure to sub-cytotoxic concentrations of the toxin could stimulate cell proliferation in the Vero-E6 line. Results showed that nanomolar concentrations of MC-LR stimulated cell cycle progression in the Vero-E6 renal cell line. Analysis of mitogen-activated protein kinases (MAPK)—specifically p38, JNK, and ERK1/2—indicates that the proliferative effect of MC-LR is mediated by the activation of this signaling cascade. To determine the effects on human vascular health, Gültekin et al. [74] analyzed how total M. aeruginosa extract and various fractions separated by chromatography influenced the viability of ECV304 endothelial cells. Different concentrations of fraction 1 either inhibited or had no effect on cell growth, while lower concentrations of fraction 3 exhibited a significant proliferative effect, ranging from 14% to 78%. Interestingly, the lowest concentration (0.047 μg/μL) produced the strongest increase in cell viability, indicating that fraction 3 contains solely proliferative substances. However, the total extract, containing all fractions, inhibited cell proliferation in a dose-dependent manner. This suggests that the toxic components in the total extract act synergistically to counterbalance the proliferative substances. A similar proliferative effect of water samples from Bulgaria was observed in human intestinal cells (HIEC-6). An MTT assay was performed on 16 water samples collected in August 2019 from water bodies with blooms. Three samples strongly stimulated cell proliferation, with increases of 194% at lower doses and 159% at higher doses [75]. The authors hypothesized that this cytoproliferative effect may be linked to potential tumor initiation, representing a step in carcinogenesis connecting consumption of freshwater from cyanobacterial bloom events with higher cancer incidence in regions where such events are frequent. The impact of purified CYN on the viability of HIEC-6 cells was also examined, revealing a clear dose-dependent reduction in cell survival [76]. In a broader evaluation across multiple cell lines, Froscio et al. [77] observed that CYN induced delayed toxicity in Vero cells. This specific line exhibited an intermediate sensitivity—appearing less susceptible than hepatic cells, yet more sensitive than the intestinal Caco-2 line. Results from in vitro cytotoxicity tests of cylindrospermopsin, pointing out exact doses and exposure duration, are summarized in Table 2.

Morphological analysis surpasses simple cytotoxicity testing in informative value because it characterizes the specific nature and localization of the resulting cellular injury. These studies are based on assessing changes in cell morphology, which may involve alterations in cell shape or structural modifications. Damage to various cellular organelles, such as membranes, mitochondria, endoplasmic reticulum, and the nucleus, is investigated. Menezes et al. [83] explored the underlying mechanisms of MC-LR-induced damage in Vero-E6 and human liver cancer (HepG2) cells, focusing specifically on the interplay between endoplasmic reticulum (ER) dysfunction and the induction of autophagy. The results showed that after 24 h of exposure, HepG2 cells were more sensitive to MC-LR compared to Vero cells. Autophagy was induced in both cell lines as a survival response at low toxin concentrations. Furthermore, MC-LR caused a concentration-dependent decrease in GRP94 expression in HepG2 cells, while no changes were observed in Vero cells. This suggests the involvement of the ER in MC-LR-induced apoptosis in HepG2 cells, whereas in kidney cells ER disruption may result from cytoskeleton-related damage. Dias et al. [84] used the micronucleus assay to examine the genotoxicity of MC-LR in kidney (Vero-E6), liver (HepG2) cell lines, and mouse leukocytes. Exposure to MC-LR at concentrations of 5 and 20 μM induced a significant increase in micronuclei in both cell lines. Additionally, analysis of micronucleus content in HepG2 cells suggested that MC-LR induces both chromosome breaks and losses. Interestingly, Miguéns and Valério [85] investigated the effects of three of the most common microcystin variants on other multicellular organisms coexisting with freshwater cyanobacteria. The results showed that MC-LR, MC-RR, and MC-YR could reduce the growth of certain heterotrophic bacteria isolated from freshwater sources.

### 2.7. Machine Learning Models

Machine learning (ML) represents a sophisticated tool for the proactive forecasting of harmful algal blooms (HABs). By integrating multi-parametric datasets and complex temporal trends, these models can generate predictive warnings. For instance, Park et al. [86] utilized eleven environmental variables across two distinct machine-learning architectures to evaluate their efficacy in predicting cyanobacterial bloom thresholds within the Changnyung Haman Reservoir in Southeast Korea. The predictive framework utilized environmental parameters such as total dissolved nitrogen, total dissolved phosphorus, mean air temperature, and average wind speed. Specifically, Villanueva et al. [87] leveraged a four-year dataset (2018–2021) from an Iowa lake to construct a model capable of forecasting harmful cyanobacterial blooms (HCBs) with a one-week lead time. The most effective predictors identified were *Microcystis mcyA* gene abundance, pH, dissolved organic carbon (DOC), and orthophosphate. Three distinct machine-learning architectures were trained and rigorously validated using performance metrics including accuracy, sensitivity, and specificity.

Recently, the Toxigenic Cyanobacteria Dataset (TCB-DS) was presented as a novel public dataset and compared with previously existing methods for early monitoring of cyanobacteria in the environment [88,89]. The proposed method is characterized with high accuracy and reliability compared with other well-known deep learning models. One of the main advantages of TCB-DS is its efficiency in identifying cyanobacteria with complex morphology. Additionally, it demonstrates the practical potential of deep learning in water quality management, allowing early detection of harmful cyanobacterial blooms with high reliability and accuracy.

The dynamics of climate change and the significant impact of human activity on the environment require the use of all available resources to develop accurate, reliable methods for early monitoring of cyanoprokaryotes in aquatic environments.

### 2.8. Rapid on-Site Monitoring

Rapid on-site testing for cyanotoxins aims to detect toxin contamination in drinking- and ambient-water samples, and to provide early warning of concentrations exceeding safe limits. This is a valuable strategy for rapid monitoring, enabling immediate assessment of public health risks and animal safety. These methods are laboratory-independent and use minimal equipment, making them a less time-consuming and cost-effective alternative to traditional lab methods.

The test strips, for example, are inexpensive and easy to use on-site, providing early warnings of common cyanotoxins [90]. However, studies assessing the efficiency and accuracy of these rapid tests indicate that some kits need improvement in specificity and sensitivity [91]. It is essential to reduce false-positive and false-negative results, as these can distort information about possible risks to human and animal health [92]. In most cases, studies using these tests will likely require further studies with more sensitive methods, such as ELISA and LC-MS [90].

Another easy-to-use on-site tool with higher sensitivity and selectivity was recently proposed [93]. The Rapid On-Site Detection of Harmful Algal Blooms (RosHAB) is a portable DNA sequencing device that enables real-time sequencing of genetic material from samples to identify toxin-producing cyanobacterial species or toxin-synthesizing genes [93,94]. The data obtained by this method provide genomic information for toxin-producing organisms and distinguishing between different cyanobacterial species and, thus, potentially predict toxin production. However, the disadvantage of such an approach is the determination only of potency of toxin contamination rather than a direct measurement of cyanotoxin molecules.

Portable biosensors are miniature devices, often based on optical, electrochemical or fluorescent approaches, which detect cyanotoxins using specific reagents (antibodies, aptamers) and provide quantitative results [95,96]. The equipment used in these devices could include fluorescent competitive immunoassays, optical planar waveguides, and nanozyme-enhanced papers connected to a smartphone for result reading [96]. The main advantage of these methods, compared to the other rapid on-site approaches, is higher sensitivity, precision and capability of quantifying toxins. However, compared to test strips, these devices need more expensive equipment and consumables. Furthermore, they are still limited with regard to the full spectrum of all cyanotoxins.

In summary, rapid on-site tests generally do not replace gold laboratory standards (e.g., ELISA and LC MS/MS), but serve as an initial screening and guidance for further analysis.

### 2.9. Satellite Monitoring

Satellite monitoring of the color of waters with suspected cyanobacterial blooms is based on remote quantifying of cyanobacterial pigments, and allows for a remote characterization of the seasonal occurrence of the blooms. Typically, the identification and quantification of cyanobacteria is based on measuring chlorophyll-a (Chl-a), which indicates total phytoplankton biomass and phycocyanin (PC), a unique accessory pigment strongly correlating with the biomass in cyanobacteria [97].

Satellite monitoring is valuable for evaluating cyanobacterial biomass, but not the amount of toxins in the water [90].Photobioreactors for the Monitoring of Cyanobacteria are created based on in vivo fluorescence spectroscopy [98], where changes in autofluorescence of cyanobacterial cultures are detected to provide primary analysis of the aquatic ecosystem behavior. Future development of devices based on this methodology would provide information beyond optical density measurements on the behavior of the aquatic populations of toxogenic cyanobacteria.

## 3. Key Highlights

This review explores the various methodologies used to monitor and predict toxic cyanobacterial blooms, highlighting the trade-offs between precision, speed, and cost. A 2024 study [99] compared several monitoring techniques against LC-MS/MS, which is considered the “gold standard” for accuracy (Table 3). While all methods showed significant correlation with microcystin levels, those targeting specific toxins or genes provided the most reliable results.

In terms of predictive modeling, beyond direct detection, machine learning has emerged as a tool for forecasting blooms up to two weeks in advance [100]. However, these models face significant hurdles—they require massive datasets for training, and high computational costs make them currently impractical for many real-world applications.

On top of that, the expansion of research into understudied regions experiences significant challenges. Unlike well-funded regions that utilize integrated satellite and molecular monitoring, developing countries face limited analytical infrastructure, which is expensive and requires specialized technical expertise (technological gaps in early warning, and lack of legal frameworks for monitoring cyanobacteria biomass and cyanotoxin concentrations in drinking and ambient water [101]). Moreover, subtropical and tropical climates, dominant in many developing regions, foster year-round bloom persistence. This increased duration, coupled with high anthropogenic nutrient loading, creates a continuous exposure risk that traditional seasonal monitoring fails to capture [5].

In summary, while newer technologies like ML and genotyping offer high precision and foresight, traditional methods like microscopy and satellite monitoring remain essential for biomass quantification and broad public-health warnings.

## 4. Conclusions

Protecting human and animal populations and ecosystems from the adverse effects of harmful cyanobacterial blooms requires proactive early warning systems.

With the global rise in bloom intensity, transitioning from reactive management to sophisticated, predictive monitoring is vital for ecosystem resilience. Even in the post-bloom period, the presence of toxins in the environment poses a danger to animal and human health, which makes their establishment in the environment even more urgent. Assessing cyanotoxin contamination in water and food, particularly in developing countries, still requires further research, including studies aimed at the simultaneous detection of multiple cyanotoxin types [102]. As established in this review, a diverse array of methodologies is available for the detection of cyanobacterial toxins, each characterized by specific technical strengths and constraints. Consistently, reported toxin concentrations surpass the World Health Organization’s provisional tolerable daily intake, signaling a persistent risk to public health. However, a critical limitation of current early warning systems remains: most analytical methods provide a snapshot of existing toxicity, without the predictive capacity to forecast future toxicological developments. Nevertheless, the early detection of cyanotoxins remains valuable and sufficiently timely to mitigate adverse impacts on public health and the economy. Methods that have demonstrated potential for predicting future toxic algal blooms include PCR-based approaches such as qPCR and machine learning models, which allow the integration of multiple variables for effective early warning and risk management.

## Figures and Tables

**Table 1:** ELISA method used to detect cyanotoxins.

Cyanotoxin	Extraction/Sample Preparation	Detected Amount	Location	Reference
Microcystin, Nodularins	Filtration, centrifugation, dilution, freeze–thaw pre-treatment	0.21–1.05 μg/L	Lake water, river water, tap water, China	Liu et al., 2023 [31]
Microcystin-LR	Vacuum filtration, simple freeze–thaw, centrifugation	0.63 ± 0.3 μg/L	River water, USA	Woodruff et al., 2024 [32]
Microcystins, Nodularin	Three freeze–thaw cycles, filtration	Not specified	Drinking water, USA	Adams et al., 2025 [33]
Microcystins	Filtration, freeze–thaw pre-treatment	0.68–2.58 μg/L	Reservoir water, lake water, China	Fan et al., 2022 [34]
Saxitoxins	Homogenization, filtration	0.35–215.57 μg/100g	Clams from the Alaskan Arctic	Charapata et al., 2025 [35]
Saxitoxins	Homogenization, filtration	30.5 µg/100 g	Seabirds, Gulf of Alaska	Van Hemert et al., 2020 [36]
Microcystins	Microwave treatment	0.3–2.0 μg/L	Drinking water, ambient water (Lake Erie) USA	Li et al., 2020 [37]
Saxitoxins	Not specified	0.59 µg/L	Benthic algae from lake water (Germany)	Bauer et al., 2023 [26]

**Table 2:** In vitro cytotoxicity studies conducted with cylindrospermopsin.

Experimental Model	Method	Experimental Conditions	Results	Reference
^2^ HepG2; ^3^ Caco-2	MTT test ^1^	0.4–66 μM, 1, 2, 4, 6, 24 h exposure	IC50 is 1.5 μM for HepG2 at 24 h exposure. IC50 is 6.5 μM for Caco-2 at 24 h exposure	Froscio et al., 2009 b [78]
^4^ CHO-K1	^5^ FITC analysis	0.1–10 μM, 12, 18, 24, 48 h exposure	Apoptosis at concentrations 1–2 μM and 12 h exposure. Necrosis at concentrations 5–10 μM and 24–48 h exposure	Gacsi et al., 2009 [79]
^6^ THP-1 and ^7^ Jurkat cells	MTT test	0.001–10 μM, 24, 48 h exposure	IC50 were 5.2 μM for Jurkat cells for 24 h and 2.32 μM for 48 h. In both cells, the exposure to CYN caused a viability decrease from 1 to 10 μM	Casas-Rodríguez et al., 2023 [80]
^8^ SH-SY5Y	MTT test	1–10 μg CYN/mL 24, 48 h exposure	IC50 were 1.11 μg/mL for 24 h and 0.69 μg/mL for 48 h. Cell viability decreases upon treatment with 5 μg CYN/mL	Hinojosa et al., 2023 [81]
^9^ hERα-HeLa-9903	Cell Counting Kit- 8 and MTS assays	MC-LR 20–200 μM and CYN 0.5–3 μM 24 h exposure	The highest tested concentration of MC-LR (200 μM) significantly reduces cell viability. For CYN, a significant decrease in viability is observed at 2.5 and 3 μM	Casas-Rodríguez et al., 2025 [82]

^1^ MTT–3-(4,5-Dimethylthiazol-2-yl)-2,5-Diphenyltetrazolium Bromide; ^2^ HepG2—human liver cancer cells; ^3^ Caco-2—colon cells; ^4^ CHO-K1—Chinese hamster ovary cells; ^5^ FITC analysis—Fluorescein Isothiocyanate analysis; ^6^ THP-1—human leukemia monocytic cell line; ^7^ Jurkat cells—human T-lymphocyte cell line; ^8^ SH-SY5Y—human neuroblastoma cell line; ^9^ hERα-HeLa-9903—human cervical cancer cell line.

**Table 3:** Comparison of detection methods.

Method	Primary Data Provided	Key Advantages/Uses
LC-MS/MS	Precise toxin concentration	The “gold standard” for microcystin detection.
ELISA	Toxin presence/levels	Strong correlation with gold standard; toxin-specific.
qPCR (Genotyping)	Precision taxonomy and toxin genes	Identifies the genetic potential for toxin production.
Microscopy	Cell counts and taxonomy	Predicts toxin type and quantifies biomass, via counting.
Fluorometry	Life cycle and ecosystem data	Tracks cyanobacteria behavior within the aquatic system.
Satellite Monitoring	Biomass density	Serves as a public health early-warning system.

## Data Availability

No new data were created or analyzed in this study. Data sharing is not applicable to this article.

## Abbreviations

ALT	Alanine Aminotransferase
AST	Aspartate Aminotransferase
ATX	Anatoxin-a
Caco-2	Colon Cells
CAT	Catalase
CHO-K1	Chinese Hamster Ovary Cells
CYN	Cylindrospermopsins
CYP	Cytochrome P450
DA	Domoic Acid
ECV304	Human Endothelial Cells
ELISA	Enzyme-Linked Immunosorbent Assay
ER	Endoplasmic Reticulum
FITC	Fluorescein Isothiocyanate
FP	Fluorescent Polarization
FPIA	Fluorescence Polarization Immunoassay
GPX1	Glutathione Peroxidase 1
GSH	Glutathione
GST	Glutathione S-Transferase
HEK293	Human Embryonic Kidney 293 Cells
HepG2	Human Liver Cancer Cells
HIEC-6	Human Intestinal Cells
MC	Microcystins
ML	Machine Learning
MTT	3-(4,5-Dimethylthiazol-2-yl)-2,5-Diphenyltetrazolium Bromide
NOD	Nodularins
OA	Okadaic Acid
PCR	Polymerase Chain Reaction
PP	Protein Phosphatases
PPIA	Protein Phosphatase Inhibitory Assay
qPCR	quantitative Polymerase Chain Reaction
ROS	Reactive Oxygen Species
SOD	Superoxide Dismutase
STX	Saxitoxin
Vero-E6	Renal Cell Line
WHO	Word Health Organization
